# Ratio of venous-to-arterial PCO_2_ to arteriovenous oxygen content difference during regional ischemic or hypoxic hypoxia

**DOI:** 10.1038/s41598-021-89703-5

**Published:** 2021-05-13

**Authors:** Jihad Mallat, Benoit Vallet

**Affiliations:** 1 Department of Critical Care Medicine, Critical Care Institute, Cleveland Clinic Abu Dhabi, Al Maryah Island, Abu Dhabi, UAE; 2 Cleveland Clinic Lerner College of Medicine of Case Western Reserve University, Cleveland, OH USA; 3 Normandy University, UNICAEN, ED 497, Caen, France; 4 EA2694, University of Lille, Lille, France

**Keywords:** Medical research, Preclinical research

## Abstract

The purpose of the study was to evaluate the behavior of the venous-to-arterial CO_2_ tension difference (ΔPCO_2_) over the arterial-to-venous oxygen content difference (ΔO_2_) ratio (ΔPCO_2_/ΔO_2_) and the difference between venous-to-arterial CO_2_ content calculated with the Douglas’ equation (ΔCCO_2D_) over ΔO_2_ ratio (ΔCCO_2D_/ΔO_2_) and their abilities to reflect the occurrence of anaerobic metabolism in two experimental models of tissue hypoxia: ischemic hypoxia (IH) and hypoxic hypoxia (HH). We also aimed to assess the influence of metabolic acidosis and Haldane effects on the PCO_2_/CO_2_ content relationship. In a vascularly isolated, innervated dog hindlimb perfused with a pump-membrane oxygenator system, the oxygen delivery (DO_2_) was lowered in a stepwise manner to decrease it beyond critical DO_2_ (DO_2crit_) by lowering either arterial PO_2_ (HH-model) or flow (IH-model). Twelve anesthetized and mechanically ventilated dogs were studied, 6 in each model. Limb DO_2_, oxygen consumption (${\dot{\text{V}}\text{O}}_{2}$), ΔPCO_2_/ΔO_2_, and ΔCCO_2D_/ΔO_2_ were obtained every 15 min. Beyond DO_2crit_, ${\dot{\text{V}}\text{O}}_{2}$ decreased, indicating dysoxia. ΔPCO_2_/ΔO_2_, and ΔCCO_2D_/ΔO_2_ increased significantly only after reaching DO_2crit_ in both models. At DO_2crit_, ΔPCO_2_/ΔO_2_ was significantly higher in the HH-model than in the IH-model (1.82 ± 0.09 vs. 1.39 ± 0.06, *p* = 0.002). At DO_2crit_, ΔCCO_2D_/ΔO_2_ was not significantly different between the two groups (0.87 ± 0.05 for IH vs. 1.01 ± 0.06 for HH, *p* = 0.09). Below DO_2crit_, we observed a discrepancy between the behavior of the two indices. In both models, ΔPCO_2_/ΔO_2_ continued to increase significantly (higher in the HH-model), whereas ΔCCO_2D_/ΔO_2_ tended to decrease to become not significantly different from its baseline in the IH-model. Metabolic acidosis significantly influenced the PCO_2_/CO_2_ content relationship, but not the Haldane effect. ΔPCO_2_/ΔO_2_ was able to depict the occurrence of anaerobic metabolism in both tissue hypoxia models. However, at very low DO_2_ values, ΔPCO_2_/ΔO_2_ did not only reflect the ongoing anaerobic metabolism; it was confounded by the effects of metabolic acidosis on the CO_2_–hemoglobin dissociation curve, and then it should be interpreted with caution.

## Introduction

In a landmark study, Vallet et al. demonstrated the determinant role of blood flow in the tissue hypoxia-induced increased venous-to-arterial CO_2_ tension difference (∆PCO_2_)^1^. Their data supported the hypothesis that increases in the venous PCO_2_ are primarily a function of changes in regional blood flow, independently of the degree of hypoxia. Gutierrez G has confirmed this conclusion in a mathematical model of tissue-to-blood CO_2_ exchange during hypoxia^2^. In these previous publications, the behavior of ∆PCO_2_ over the arterial-to-venous oxygen content difference (ΔO_2_) ratio (ΔPCO_2_/ΔO_2_), and the difference between venous-to-arterial CO_2_ content (ΔCCO_2_) over ΔO_2_ ratio (ΔCCO_2_/ΔO_2_) in a model of progressive tissue hypoxia generated by reducing either flow [ischemic hypoxia (IH)] or arterial oxygen tension [hypoxic hypoxia (HH)], were not investigated^1,2^.

Several clinical studies^3–7^ have shown that ΔPCO_2_/ΔO_2_ ratio, taken as a surrogate of respiratory quotient (RQ), was associated with elevated lactate levels and oxygen supply dependency considered, in those studies, as indices of global anaerobic metabolism in critically ill patients with tissue hypoperfusion. However, in an experimental study, Dubin et al. found that ΔPCO_2_/ΔO_2_ ratio was a poor indicator of anaerobic metabolism in the hemodilution model of tissue hypoxia, where anemia was associated with preserved blood flow^8^. Similarly, other authors suggested that ΔPCO_2_/ΔO_2_ ratio might not rise during tissue hypoxia conditions when associated with normal/high blood flow because venous blood flow seemed to guarantee a sufficient clearance of CO_2_ generated by the anaerobic metabolism^9^. Thus, it is unclear if the ΔPCO_2_/ΔO_2_ ratio would be able to depict the presence of anaerobic metabolism in patients with maintained blood flow (cardiac output).

Furthermore, one estimates that the ΔPCO_2_/ΔO_2_ ratio might be affected by other factors than anaerobic metabolism by influencing the relationship between CO_2_ content (CCO_2_) and PCO_2_. Indeed, metabolic acidosis can change the PCO_2_/CCO_2_ relationship so that PCO_2_ is higher for a given CCO_2_. Low oxygen saturation, by promoting more CO_2_ binding to hemoglobin (Haldane effect), increases the CCO_2_ for a given PCO_2_^10^. It is not completely clear to what extent these factors would impact the PCO_2_/CCO_2_ relationship and influence the ΔPCO_2_/ΔO_2_ ratio. Answering this question would help to define the applicability of this ratio in different clinical situations.

Therefore, we used, in secondary analysis, the original study published by Vallet et al.^1^ with the aim to assess the behavior of ΔPCO_2_/ΔO_2_ ratio, ΔCCO_2_/ΔO_2_ ratio, and their components in the regional model of progressive tissue hypoxia generated by IH or HH^1^. We also investigated the metabolic acidosis (pH) and Haldane effects on the PCO_2_/CCO_2_ relationship. Since the flow was maintained unchanged in the HH model, we hypothesized that ΔPCO_2_/ΔO_2_ and ΔCCO_2_/ΔO_2_ ratios might not be able to detect the occurrence of anaerobic metabolism as the sustained blood flow would be sufficient to wash out the CO_2_ generated by hypoxic cells in that model.

## Methods

### Animal preparation

The original study was approved by the University of Alabama at Birmingham Institutional Animal Care and Use Committee. The study is reported in accordance with the ARRIVE guidelines. All experiments were performed in accordance with relevant guidelines and regulations. Twelve dogs of either sex and mixed breed were used^1^. All animals were anesthetized with intravenous 30 mg/kg of sodium phenobarbital and mechanically ventilated with a Harvard animal respirator at 10 breaths/min. Lamps suspended above the operating table were used to maintain core temperature near 37 °C. Tidal volume was varied to maintain systemic arterial PCO_2_ between 30 and 35 mmHg. The ventilator setting was kept unchanged during the rest of the experiment. A 20 mg of succinylcholine chloride was given intramuscularly and a continuous infusion (0.1 mg/mL/min) was begun. Anesthesia depth was checked regularly by vigorous toe pinching, and additional anesthetic was given if systemic blood pressure and heart rate responded.

Catheters were inserted into the pulmonary artery (through the internal jugular vein) and common carotid artery for continuous measurements of vascular pressures and blood sampling. Arterial inflow (Q) and venous outflow from the left hindlimb were isolated, as previously described^1,11^ (Supplemental Digital Content 1, Appendix). A roller occlusive pump directed blood flow from the right hindlimb femoral artery to the femoral artery of the vascularly isolated left hindlimb. A sampling port and pressure transducer were placed in this circuit proximal to the limb. A membrane oxygenator (model 0800-2A, Sci Med) was interposed in the perfusion circuit. A gas flow mixer (model GF-3, Cameron Instruments) supplied O_2_, N_2_, and CO_2_ to the oxygenator, as needed, to produce normoxia or hypoxia with normocapnia in the blood supply to the hindlimb. A water bath warmed the oxygenator so that perfusion to the isolated hindlimb was at 37 °C after heat loss through the tubing.

### Measurements

Blood samples from the carotid, femoral, and pulmonary arteries and femoral vein were obtained simultaneously. Blood gas tensions and pH were measured in an acid–base analyzer (ABL-30, Radiometer, Westlake, OH) at 37 °C and later corrected to esophageal temperature at the time of sampling. Oxygen saturation was measured with a co-oximeter calibrated for dog blood (IL-282, Instrumentation Lab, Lexington, MA). Arterial oxygen content was calculated as CaO_2_ (mL) = 1.34 × Hb (g/dL) × SaO_2_ + 0.0031 × PaO_2_ (mmHg), where SaO_2_ is the oxygen saturation of arterial blood, Hb the hemoglobin concentration, and PaO_2_ the arterial oxygen tension. Hindlimb venous oxygen content was calculated as CvO_2_ (mL) = 1.34 × Hb (g/dL) × SvO_2_ + 0.0031 × PvO_2_ (mmHg), where PvO_2_ is the hindlimb venous oxygen tension, and SvO_2_ is the hindlimb venous oxygen saturation. ΔO_2_ was calculated as CaO_2_ – CvO_2_. Hindlimb VO_2_ (${\dot{\text{V}}\text{O}}_{2}$)was calculated as the product of Q (leg blood flow) and ΔO_2_. Hindlimb oxygen delivery (DO_2_) was calculated by using the formula: DO_2_ (mL/min) = CaO_2_ × Q × 10. Hindlimb oxygen extraction (OE) was defined as: OE = ${\dot{\text{V}}\text{O}}_{2} {\text{/DO}}_{2}$.

∆PCO_2_ was calculated as the difference between the hindlimb venous carbon dioxide tension (PvCO_2_) and hindlimb arterial PCO_2_ (PaCO_2_). In the original study, the hindlimb difference between venous-to-arterial CO_2_ content (CvCO_2_ − CaCO_2_) was calculated with the McHardy equation (as proposed by Neviere et al.^12^): ΔCCO_2_ = 11.02 × [(PvCO_2_)^0.396^ − (PaCO_2_)^0.396^] − (15 − Hb) × 0.015 × (PvCO_2_ − PaCO_2_) − (95 − SaO_2_) × 0.064. However, the most used equation to calculate the blood CO_2_ content is the Douglas equation^13^, which includes pH:

$$ {\text{Blood}}\;{\text{CO}}_{{2{\text{D}}}} \;{\text{content}}\;[{\text{blood}}\;{\text{Douglas}}\;{\text{CCO}}_{2} \;({\text{mL}})] = {\text{Plasma}}\;{\text{CCO}}_{2} \times [1{-}0.0289 \times ({\text{Hb}}){/}(3.352{-}0.456 \times {\text{SO}}_{2} ) \times (8.142{-}{\text{pH}})] $$

where plasma CCO_2_ = 2.226 × S × plasma ${\text{PCO}}_{2} \times (1 + 10^{{{\text{pH}}{-}{\text{pK}}^{\prime}}} )$, CCO_2_ is CO_2_ content, SO_2_ is oxygen saturation, S is the plasma CO_2_ solubility coefficient, and pK′ is the apparent pK.

S and pK′ were calculated as follow:

$$ {\text{S}} = 0.0307 + [0.00057 \times (37{-}{\text{T}})] + [0.00002 \times (37{-}{\text{T}})^{2} ] $$

and

$$ {\text{pK}}^{\prime} = 6.086 + [0.042 \times (7.4{-}{\text{pH}})] + \,((38{-}{\text{T}}) \times \{ 0.00472 + [0.00139 \times (7.4{-}{\text{pH}})]\} ) $$

where T is the temperature expressed as °C.

The difference between venous-to-arterial CCO_2_ calculated with the Douglas equation was: ΔCCO_2D_ = CvCO_2D_ − CaCO_2D_.

To investigate the metabolic acidosis and Haldane effects on the PCO_2_/CCO_2_ relationship, default (Def) values of blood CCO_2_ were calculated with the Douglas’s equation by using only the resting values of pH and SvO_2_ for each dog as following: DefpH-ΔCCO_2D_ = DefpH-CvCO_2D_ − DefpH-CaCO_2D_, and DefSvO_2_-ΔCCO_2D_ = DefSvO_2_-CvCO_2D_ − DefSvO_2_-CaCO_2D_.

Leg blood flow, DO_2_, and ${\dot{\text{V}}\text{O}}_{2}$ were reported per kilogram of muscle mass.

We also calculated the hindlimb ΔPCO_2_/ΔO_2_, ΔCCO_2_/ΔO_2_, and ΔCCO_2D_/ΔO_2_ ratios_._

### Experimental protocol

The experimental model was already described previously^1^. After all pressures and flows were stable for at least 30 min, the experiment began with a 30-min control period, during which measurements were obtained every 15 min. In the progressive ischemic hypoxia (IH) group, Q was then decreased every 15 min to produce Q values of ~ 60, 45, 40, 30, 20, 15, and 10 mL/kg/min. In the hypoxic hypoxia (HH) group, Q was set at 60 mL/kg/min and limb DO_2_ was reduced by decreasing arterial PO_2_ from 100 to ~ 15 mmHg (i.e., CaO_2_ of 17 to 2 mL O2/100 mL) in eight steps at 15-min intervals. A flow rate of 60 mL/kg/min was chosen for progressive hypoxia because it is within the range of resting blood flow to normal skeletal muscle and for the practical reason that a moderate flow was necessary to achieve the desired low PO_2_ values using the membrane oxygenator. Oxygen and CO_2_-derived variables were determined every 15 min, 13 min after the change in hindlimb arterial flow or PO_2_.

For each experiment, regression lines were fitted to the delivery independent and dependent portions of the delivery-uptake curve using a dual-line, least squares method^14^. The intercept of these two lines defined the critical DO_2_ (DO_2_crit), that is, the delivery at which ${\dot{\text{V}}\text{O}}_{2}$ began to fall with any further decline in DO_2_.

### Statistical analysis

All data are expressed as mean ± SEM after assessed for normality using the Kolmogorov–Smirnov test.

Comparisons of data within and between groups were performed using a mixed ANOVA. Post-hoc paired and unpaired *t* tests were used, as appropriate, for one-time comparisons. The Bonferroni method was used to adjust for multiple comparisons.

Statistical analysis was performed using GraphPad Prism 6.0 software for windows (San Diego, California, USA). *p* < 0.006 and *p* < 0.007 were considered statistically significant for the between-group and within-group (with the baseline) comparisons, respectively. All reported *p* values are two-sided.

## Results

Systemic hemodynamics and oxygen-derived variables remain unchanged throughout the protocol with no differences between the IH and HH models (Supplemental Digital Content 2, Table S1).

In both groups, the ${\dot{\text{V}}\text{O}}_{2}$/DO_2_ graph depicts the typical biphasic relationship (Supplemental Digital Content 3, Figure S1). There was no statistically significant difference between the mean DO_2Crit_ in the HH and IH models (6.9 ± 0.6 vs. 6.0 ± 0.5 mL/kg/min, *p* = 0.28, respectively). SvO_2_ at DO_2Crit_ was not statistically different between the two groups (25 ± 1.7% in HH vs. 26 ± 1.5% in IH, *p* = 0.66). However, for the lower DO_2_ values, SvO_2_ was significantly higher in the IH model than in the HH group (Supplemental Digital Content 4, Figure S2). EO_2_ at DO_2Crit_ was significantly higher in the IH group than in the HH model (74 ± 2% vs. 60 ± 4%, *p* = 0.01) and increased continuously and similarly in both groups (Supplemental Digital Content 5, Figure S3). ΔPCO_2_ risen significantly in the IH model and did not change in the HH model (Supplemental Digital Content 6, Figure S4).

### Time course of venous-to-arterial CCO_2_ difference

**Figure 1:**
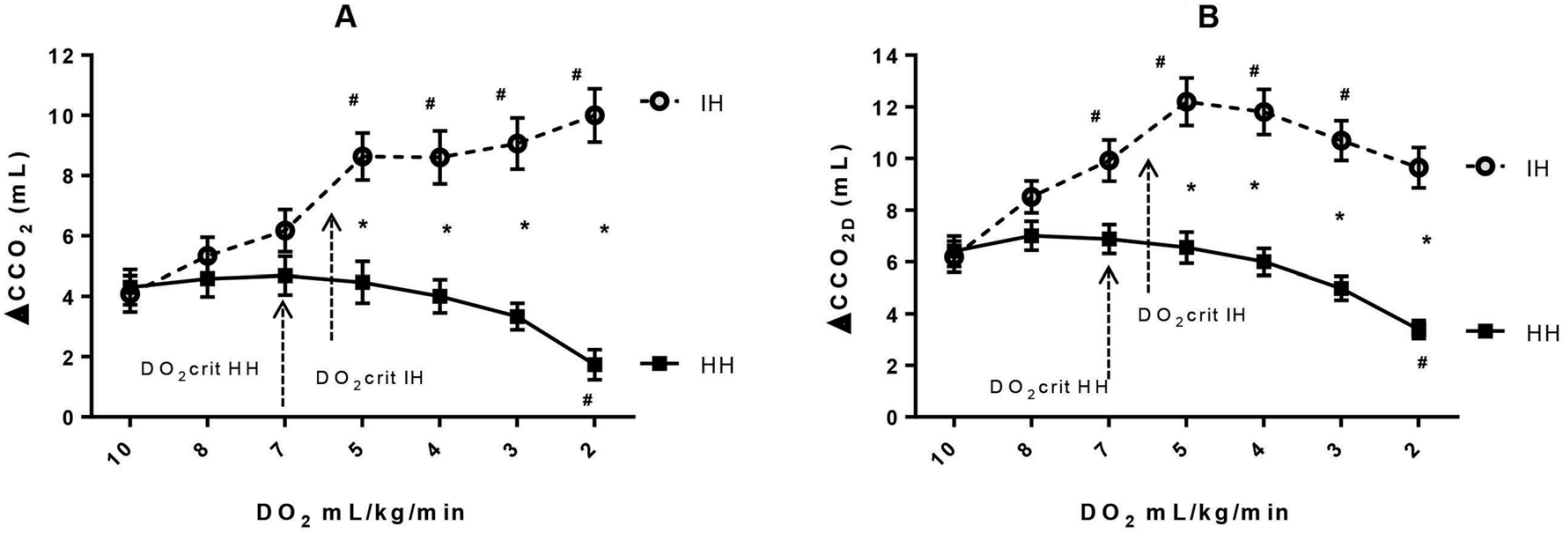
Hindlimb venous-to-arterial CO_2_ content difference (ΔCCO_2_) calculated with McHardy equation (**A**) and with Douglas equation  (ΔCCO_2D_) (**B**) as a function of hindlimb oxygen delivery (DO_2_) for ischemic hypoxia model (IH) and hypoxic hypoxia model (HH). *p < 0.006 vs. HH, ^#^*p* < 0.007 vs. baseline, mixed ANOVA.

ΔCCO_2_ calculated with the McHardy equation increased progressively along with the decrease in DO_2_ in the IH group but remained unchanged and even significantly decreased at the lowest DO_2_ value on the HH group (Fig. 1A). At DO_2Crit_, ΔCCO_2_ was significantly higher in the IH group than in the HH group (7.5 ± 0.66 vs. 4.6 ± 0.5 mL, *p* = 0.006, respectively), and it was significantly different from the baseline only in the IH group (*p* = 0.0023).

ΔCCO_2D_ calculated with the Douglas equation, in the IH group, increased with the decrease in DO_2_ down to DO_2crit_. However, beyond DO_2crit_, ΔCCO_2D_ started to decrease with the further decline in DO_2_ to become not significantly different from its baseline value at the lowest value of DO_2_ (Fig. 1B). In the HH group, ΔCCO_2D_ had the same pattern as ΔCCO_2_ calculated with the McHardy equation (Fig. 1A,B), which remained unchanged in parallel with the decreases in DO_2_ to become significantly lower than its baseline (*p* < 0.001) only at the end of the experiment. At DO_2crit_, ΔCCO_2D_ was greater in the IH group compared to the HH group (11.0 ± 0.88 vs. 7.0 ± 0.56 mL, *p* = 0.003, respectively), and it was significantly higher than its baseline value (*p* < 0.001) only in the IH group (Fig. 1B).

### pH and Haldane effects on the PCO_2_/CCO_2_ relationship

**Figure 2:**
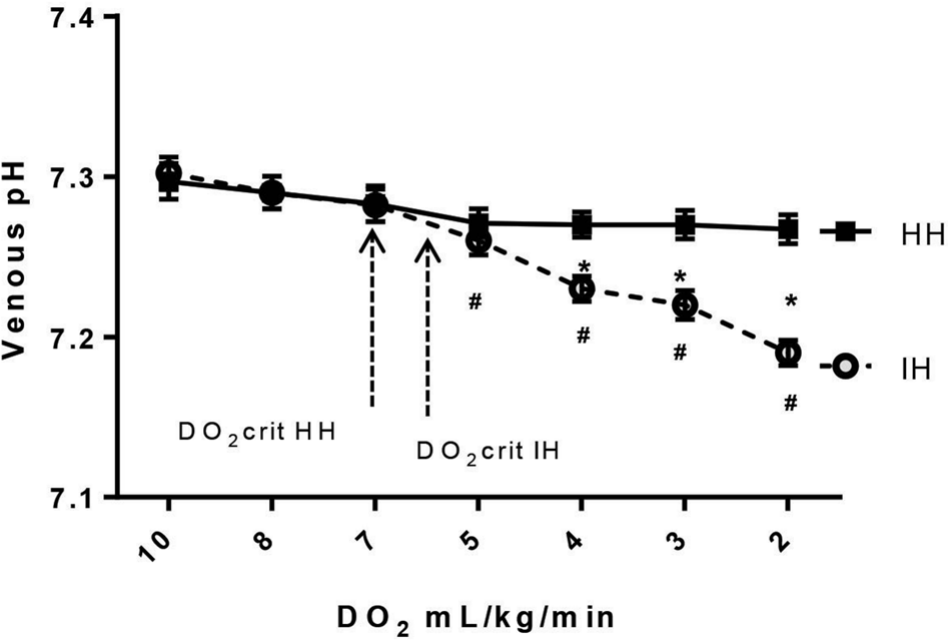
Hindlimb venous pH as a function of hindlimb oxygen delivery (DO_2_) for ischemic hypoxia model (IH) and hypoxic hypoxia model (HH). *p < 0.006 vs. HH, ^#^*p* < 0.007 vs. baseline, mixed ANOVA.

Hindlimb venous pH (pHv) remained unchanged with the decline in DO_2_ down to DO_2crit_ in both groups (Fig. 2). However, beyond DO_2crit_, pHv decreased significantly only in the IH group and remained stable in the HH group (Fig. 2).

**Figure 3:**
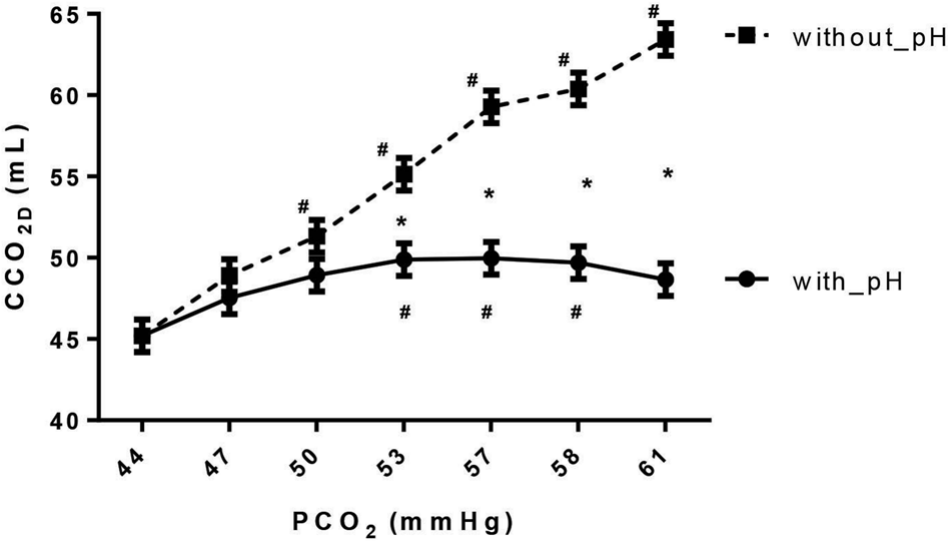
Hindlimb venous CO_2_ content (CCO_2_) as a function of hindlimb venous PCO_2_ for CCO_2_ calculated with accounting for pH changes (with_pH) and without accounting for pH changes (without_pH) using Douglas equation (CCO_2D_). *p < 0.006 vs. HH, ^#^*p* < 0.007 vs. baseline, mixed ANOVA.

**Figure 4:**
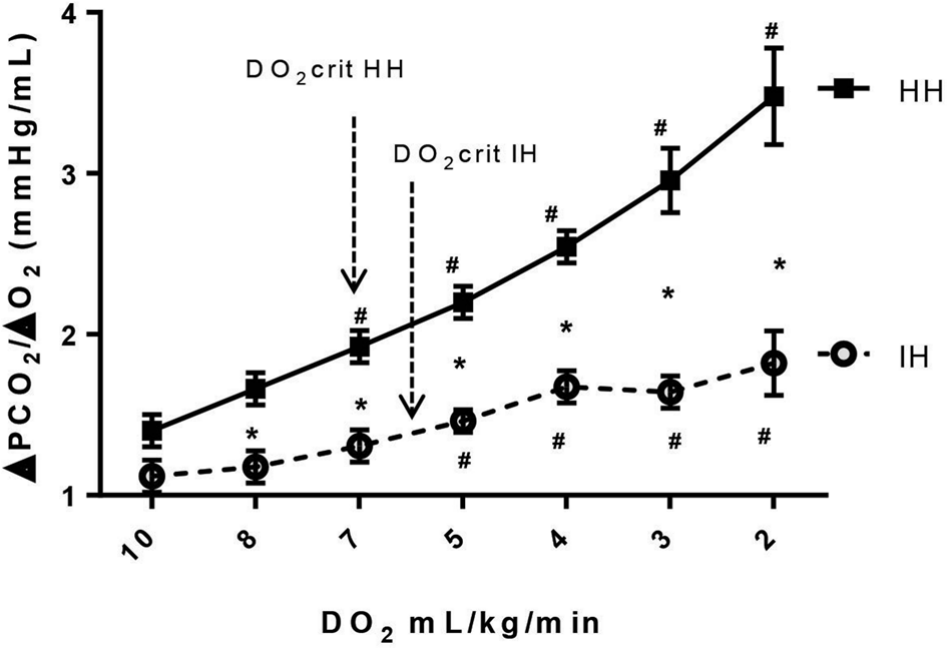
Hindlimb venous-to-arterial PCO_2_ difference (ΔPCO_2_) over the arterial-to-venous O_2_ difference (ΔO_2_) ratio (ΔPCO_2_/ΔO_2_) as a function of hindlimb oxygen delivery (DO_2_) for ischemic hypoxia model (IH) and hypoxic hypoxia model (HH). At DO_2_crit, ΔPCO_2_/ΔO_2_ was significantly higher in HH model (1.82 ± 0.09) than IH model (1.39 ± 0.06). *p < 0.006 vs. HH, ^#^*p* < 0.007 vs. baseline, mixed ANOVA.

The venous CCO_2_ calculated, with the Douglas equation, by acknowledging the changes in pHv (CvCO_2D_) increased first with the rise in PvCO_2_, but then after, it stabilized despite further increases in PvCO_2_, due to the fall in pHv. Eventually, despite the continuously increasing PvCO_2_, CvCO_2D_ decreased due to the marked decline in pHv (Fig. 3). On the contrary, there was almost a linear increase in DefpH-CvCO_2D_ (without accounting for the changes in pHv) with the increase in PvCO_2_ (Fig. 4). Also, DefpH-ΔCCO_2D_ increased linearly with the decreases in DO_2_ in the IH group, while it remained unchanged in the HH group (Supplemental Digital Content 7, Figure S5).

The relationship between PvCO_2_ and CCO_2_ calculated without accounting for the changes in SvO_2_ was the same as that if we acknowledged the variations in SvO_2_ (Supplemental Digital Content 8, Figure S6).

### Time course of ΔPCO_2_/ΔO_2_, ΔCCO_2_/ΔO_2_, and ΔCCO_2D_/ΔO_2_ ratios

ΔO_2_ increased significantly in the IH and decreased in the HH in parallel with the decreases in DO_2_ (Supplemental Digital Content 9, Figure S7).

At DO_2crit_, ΔPCO_2_/ΔO_2_ ratio was significantly higher in the HH group than in the IH group (1.82 ± 0.09 mmHg/mL vs. 1.39 ± 0.06 mmHg/mL, *p* = 0.002, respectively). In both groups, ΔPCO_2_/ΔO_2_ ratio increased significantly only after reaching DO_2crit_ (Fig. 4). Also, the increase in ΔPCO_2_/ΔO_2_ ratio was significantly higher in the HH than in the IH group.

ΔCCO_2_/ΔO_2_ ratio increased after DO_2crit_ was reached in both groups, with a trend to decrease by the end of the experiment in the HH group (Supplemental Digital Content 10, Figure S8). At DO_2crit_, there was no significant difference between the two groups (IH: 0.59 ± 0.02 vs. HH: 0.67 ± 0.03, *p* = 0.05).

**Figure 5:**
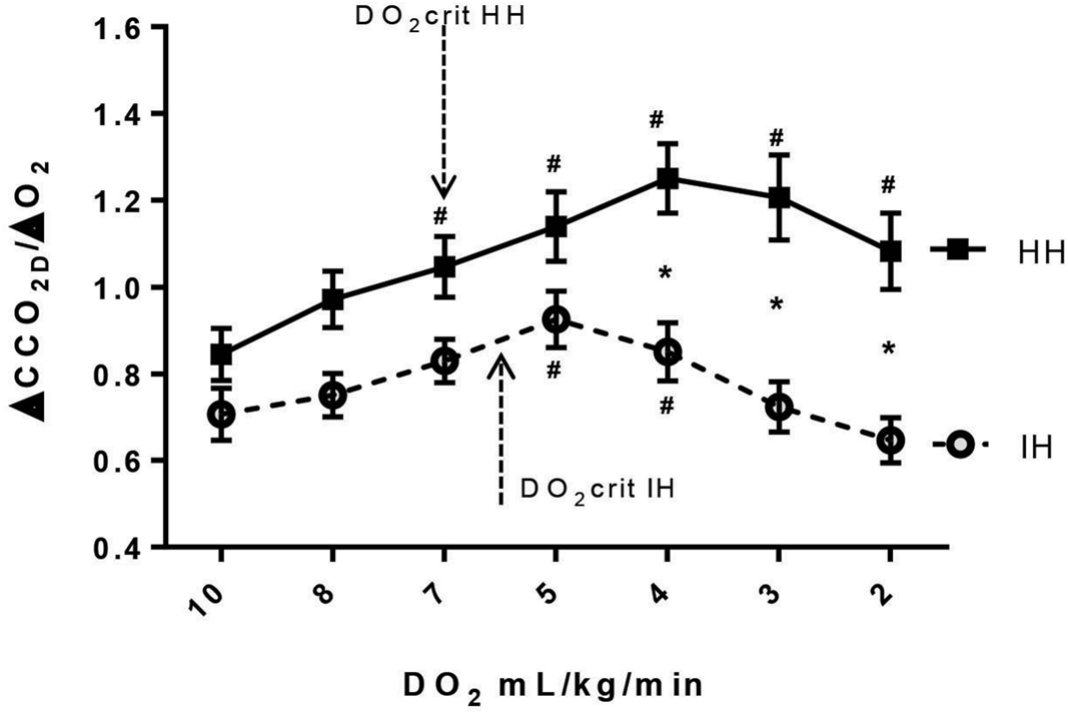
Hindlimb venous-to-arterial CO_2_ content difference calculated with Douglas equation (ΔCCO_2D_) over the arterial-to-venous O_2_ difference (ΔO_2_) ratio (ΔCCO_2D_/ΔO_2_) as a function of hindlimb oxygen delivery (DO_2_) for ischemic hypoxia model (IH) and hypoxic hypoxia model (HH). At DO_2_crit, there was no significantly difference between HH model (1.01 ± 0.06) and IH model (0.87 ± 0.05). *p < 0.006 vs. HH, ^#^*p* < 0.007 vs. baseline, mixed ANOVA.

In both groups, ΔCCO_2D_/ΔO_2_ ratio increased significantly after reaching DO_2crit_. However, in the HH group, at lower values of DO_2_, ΔCCO_2D_/ΔO_2_ ratio started to decline but remained significantly higher than its baseline value. In the IH group, beyond DO_2crit_, ΔCCO_2D_/ΔO_2_ ratio begun to decrease at a higher value of DO_2_ than in the HH group, to become not significantly different from its baseline value at the end of the experiment (Fig. 5). At DO_2crit_, ΔCCO_2D_/ΔO_2_ was not significantly different between the two groups (0.87 ± 0.05 for IH vs. 1.01 ± 0.06 for HH, *p* = 0.09).

In both groups, DefpH-ΔCCO_2D_/ΔO_2_ (without accounting for pH changes) increased similarly and linearly in parallel with the decrease in DO_2_ (Supplemental Digital Content 11, Figure S9). The increase in DefpH-ΔCCO_2D_/ΔO_2_ in IH occurred before reaching DO_2crit_.

## Discussion

The main findings of our study were that: (1) in both groups, ΔPCO_2_/ΔO_2_ as well as ΔCCO_2_/ΔO_2_, and ΔCCO_2D_/ΔO_2_ increases significantly in parallel with the decreases in DO_2_ only after reaching DO_2crit_; (2) beyond DO_2crit_, the time course of ΔPCO_2_/ΔO_2_ ratio was different from that of ΔCCO_2D_/ΔO_2_ or ΔCCO_2_/ΔO_2_ ratio, in both groups; (3) metabolic acidosis, but not Haldane effect influenced significantly the PCO_2_/CCO_2_ relationship explaining the discrepancy between ∆PCO_2_ and ΔCCO_2D_; (4) the method of CCO_2_ calculation had a considerable impact on the results and yielded different conclusions.

Anaerobic metabolism occurrence is usually due to cellular hypoxia^15^. Whenever oxygen delivery decreases relative to demand, and the compensatory mechanism is exhausted, extra-mitochondrial anaerobic glycolysis occurs, and lactic acidosis develops^16^. We aimed to investigate if ΔPCO_2_/ΔO_2_ and ΔCCO_2D_/ΔO_2_ could reflect the development of anaerobic metabolism in two regional models of tissue hypoxia: IH, where the oxygen delivery progressively decreased by decreasing the blood flow, and HH, where the blood flow was maintained unchanged, and the oxygen delivery was reduced by decreasing the arterial oxygen content.

In experimental conditions of tissue hypoxia, the drop in VO_2_ leads to decreased total VCO_2_ generation, mainly related to the decrease in aerobic CO_2_ production. However, under situations of hypoxia, tissue CO_2_ increases as hydrogen ions generated by anaerobic sources of energy (hydrolysis of high-energy phosphates) are buffering by bicarbonate existing in the cells (anaerobic CO_2_ production)^17^. Therefore, VCO_2_ being reduced less than VO_2_, the RQ (VCO_2_/VO_2_) should increase. Accordingly, the increase in RQ has been shown to be a useful marker of global tissue hypoxia^18,19^. Indeed, Groeneveld et al.^18^ observed, in an experimental model of a graded increase in positive end-expiratory pressure-induced a decrease in cardiac output and oxygen delivery in pigs, that the decline in VCO_2_ (by 21 ± 2%) was less than in VO_2_ (by 27 ± 2%).

However, airway RQ measurement necessitates a specific monitoring device (indirect calorimetry) that many hospitals might not have. Recently, there has been a growing interest in the ΔPCO_2_/ΔO_2_ ratio as a surrogate of the RQ to detect the development of global anaerobic metabolism in critically ill patients^3–7^. Indeed, several studies found an association between increased ΔPCO_2_/ΔO_2_ ratio and hyperlactatemia^5^ and decreased lactate clearance^6,7^, which were taken as markers of anaerobic metabolism activation. We^4^ and other authors^3^ have also shown that ΔPCO_2_/ΔO_2_ ratio had an excellent ability to detect the presence of VO_2_/DO_2_ dependency phenomenon, better than central venous oxygen saturation and blood lactate levels, in septic shock patients. Recently, Mesquida et al.^20^ reported an association between ΔPCO_2_/ΔO_2_ ratio and ICU mortality in septic shock patients. In contrast, in other studies, ΔPCO_2_/ΔO_2_ was unable to predict hyperlactatemia, poor lactate clearance, or VO_2_/DO_2_ dependency and was not associated with outcome in septic shock or cardiac surgery patients^9,21–23^. Thus, the relationship between ΔPCO_2_/ΔO_2_ and the presence of tissue hypoxia is controversial.

Indeed, the use of ΔPCO_2_/ΔO_2_ ratio as a surrogate of RQ supposes that the PCO_2_/CCO_2_ relationship is quasi-linear, which may be true over the physiological range of PCO_2_^24^. However, this relationship can be influenced by the degree of metabolic acidosis^25^, hematocrit^26^, and oxygen saturation (Haldane effect)^8,27^, and it becomes nonlinear if these factors change^28^. Indeed, severe metabolic acidosis, low hematocrit, and high oxygen saturation can increase PCO_2_ for a given CCO_2_ since less CO_2_ is bound to hemoglobin^8^. Thus, ∆PCO_2_ and ΔPCO_2_/ΔO_2_ ratio might be increased due to several factors unrelated to the blood flow and anaerobic metabolism. We found that metabolic acidosis influenced the PCO_2_/CCO_2_ relationship significantly. Indeed, when the changes in pHv were ignored, the PCO_2_/CCO_2_ relationship was almost linear (Fig. 3). However, CCO_2_ was not linearly related to PCO_2_ when the changes in pH were acknowledged. In fact, PCO_2_ and CCO_2_ changed in opposite directions as metabolic acid was added to the blood by the hypoxic cells (Fig. 3). That is because metabolic acidosis causes plasma and red blood cell CCO_2_ and bicarbonates to decrease^29^. In our study, the Haldane effect did not influence the PCO_2_/CCO_2_ relationship as the latter was the same, taking into account or not for the changes in SvO_2_ (Supplemental Digital Content 8, Figure S6).

Our findings suggest that, in situations with moderate/severe metabolic acidosis, an elevated ∆PCO_2_ might not reflect only low or inadequate blood flow but could also be ascribed to modifications of the CO_2_–hemoglobin dissociation curve. Our results are in line with previous studies. Indeed, Sun et al.^29^ found that, in healthy subjects, during heavy exercise, changes in pH had a significant influence on the PCO_2_/CCO_2_ relationship with CCO_2_ not linearly related to PCO_2_ and even varied in opposite directions after the lactic acidosis threshold was reached. However, in that study, changes in SO_2_ (Haldane effect) had a minor influence on the PCO_2_/CCO_2_ relationship. Also, in septic shock patients, Mesquida et al.^20^ observed that pH was the only best predictor of the discrepancy found between ΔPCO_2_/ΔO_2_ and ΔCCO_2D_/ΔO_2_; venous oxygen saturation (Haldane effect) had a minimal effect.

We observed that ΔPCO_2_/ΔO_2_, and ΔCCO_2D_/ΔO_2_ significantly increased at DO_2crit_ and not before (Figs. 4 and 5), suggesting that these variables were able to depict the occurrence of oxygen supply dependency (DO_2crit_) in both IH and HH groups. The increases in these variables were mainly due to the decline in ΔO_2_ in the HH group and the rise in ∆PCO_2_ and ΔCCO_2_ in the IH group induced by the decrease in blood flow. In contrast, in an experimental study of hemodilution model of tissue hypoxia, Dubin et al.^8^ found that ΔPCO_2_/ΔO_2_ significantly increased before the fall in VO_2_ and the sharp increase in RQ (measured by indirect calorimetry), and thus, it was a misleading indicator of anaerobic metabolism. The authors explained this finding by the effects of low hemoglobin on the CO_2_–hemoglobin dissociation curve^8^. However, it is hard to compare these results together as the two tissue hypoxia models (HH and hemodilution) are different. Indeed, the effects of anemia on the CO_2_–hemoglobin dissociation curve could be different from that of the low oxygen saturation (Haldane effect). Also, the magnitude of the decrease in venous oxygen saturation would be much more pronounced in the HH model, where the flow was maintained constant, than in the hemodilution model, where cardiac output increased by 126%^8^. Beyond DO_2crit_, we observed a discrepancy between the evolutions of ΔPCO_2_/ΔO_2_ and ΔCCO_2D_/ΔO_2_ in both groups (Figs. 4 and 5). That might be explained by the different behavior of ∆PCO_2_ and ΔCCO_2D_ at lower DO_2_ values. Indeed, in the IH group, these two variables changed in opposite directions: ∆PCO_2_ continued to increase, whereas ΔCCO_2D_ fell caused by metabolic acidosis (decreases in bicarbonate levels). In the HH model, ∆PCO_2_ remained unchanged, whereas ΔCCO_2D_ decreased at lower DO_2_ values (Fig. 1B and Supplemental Digital Content 6, Figure S4). Therefore, below DO_2crit_, and at very low DO_2_ values, ΔPCO_2_/ΔO_2_ ratio is confounded by the changes in the CO_2_–hemoglobin curve induced by metabolic acidosis, and it does not reliably reflect the oxygen supply dependency phenomenon and the activation of anaerobic metabolism, especially in the IH tissue hypoxia model. However, in clinical practice, in such cases with very low DO_2_, the clinical diagnosis of tissue hypoxia would be obvious without the need for such markers.

It is worth to note that the method of calculation of the difference in CCO_2_ matters as the McHardy equation^12^, and Douglas equation^13^ yielded different findings (Figs. 5 and Supplemental Digital Content 10, Figure S8). However, we think that the Douglas equation is much more used in research papers, and more accurate as it accounts for much more factors such as pH.

There is no reported data, in the literature, on the behavior of ΔCCO_2D_/ΔO_2_ ratio beyond DO_2crit_ at very low DO_2_ values. This ratio tended to decrease in both tissue hypoxia models, even in the presence of anaerobic CO_2_ production. It is possible that in case of advanced tissue hypoxia with massive decreases in VO_2_, the anaerobic sources of CO_2_ becoming much less important than the dramatically decreased aerobic ones leading to a reduction in VCO_2_/VO_2_ ratio.

We acknowledge several limitations to our study. First, our study was a secondary analysis that is subject to inherent limitations. Second, computation of CCO_2_ is subject to an important potential risk of measurement errors due to the number of variables included in the equation^30^ that might amplify during the calculation of ΔCCO_2D_. Nevertheless, ΔCCO_2D_/ΔO_2_ ratio was already shown to be associated with mortality in septic shock patients^9^, suggesting that the influence of measurement errors might be limited.

## Conclusions

In both IH and HH regional models of tissue hypoxia, ΔPCO_2_/ΔO_2_ and ΔCCO_2D_/ΔO_2_ ratios both widened significantly only at the beginning of oxygen supply dependency. The hypoxic tissue hypoxia model yielded higher increases in ΔPCO_2_/ΔO_2_ than the IH model. At advanced stages of tissue hypoxia (very low DO_2_), ΔPCO_2_/ΔO_2_ did not only reflect the ongoing anaerobic metabolism, but it was confounded by the effects of metabolic acidosis on the CO_2_–hemoglobin dissociation curve, and then it should be interpreted with caution. For clinical practice, in severe metabolic acidosis situations, elevated ∆PCO_2_ may not reflect the degree of tissue hypoperfusion. In these cases, calculating the difference in CCO_2_ with the Douglas equation is advisable.

## Supplementary Information

Supplementary Information 1.

Supplementary Information 2.

Supplementary Information 3.

Supplementary Information 4.

Supplementary Information 5.

Supplementary Information 6.

Supplementary Information 7.

Supplementary Information 8.

Supplementary Information 9.

Supplementary Information 10.

Supplementary Information 11.

## Abbreviations

CO_2_: Carbon dioxide
VO_2_: Oxygen consumption
VCO_2_: Carbon dioxide production
DO_2_: Oxygen delivery
RQ: Respiratory quotient
∆PCO_2_: Venous-to-arterial carbon dioxide tension difference
CCO_2_: CO_2_ content
ΔCCO_2_: Venous-to-arterial carbon dioxide content difference
CCvCO_2_: Venous CO_2_ content
CCaCO_2_: Arterial CO_2_ content
ΔO_2_: Arterial-to-venous oxygen content difference
PaCO_2_: Partial arterial carbon dioxide tension
PvCO_2_: Partial venous carbon dioxide tension
SvO_2_: Venous oxygen saturation
SaO_2_: Arterial oxygen saturation
PaO_2_: Partial arterial oxygen tension
PvO_2_: Partial venous oxygen tension
Hb: Hemoglobin
