# Supplementary material for: Ratio of venous-to-arterial PCO2 to arteriovenous oxygen content difference during regional ischemic or hypoxic hypoxia
Source: Sci Rep. 2021 May 13;11:10172. doi: 10.1038/s41598-021-89703-5 (PMC8119496; doi:10.1038/s41598-021-89703-5)
Supplement: Supplementary file 1 — Supplementary Information 1. [file 41598_2021_89703_MOESM1_ESM.docx]

**Supplemental Digital Content 1**

**Appendix**

**Methods**

*Animal preparation*

This study was approved by the University of Alabama at Birmingham Institutional Animal Care and Use Committee. Dogs of either sex and mixed breed were used. All animals were initially anesthetized with intravenous pentobarbital sodium (30 mg/kg) and intubated with a cuffed endotracheal tube. Catheters were inserted into the pulmonary artery (via the internal jugular vein) and common carotid artery for continuous measurement of vascular pressures and blood sampling. Lamps suspended above the operating table were used to maintain core temperature near 37°C. Standard limb leads were used to obtain heart rate continuously by means of a cardiotachometer (type 9857 cardiotachometer coupler, Beckman Instruments, Schiller Park, IL).

Arterial inflow (Q) and venous outflow from the left hindlimb were isolated, as previously described [1]. In brief, the proximal 10 cm of the femoral nerve, artery, and vein were dissected free in the groin, and all vascular branches were tied off. Venous outflow from the limb was restricted to the femoral vein by tourniquet technique. With the use of a spinal needle as an introducer, a nylon cord was passed through the limb on each side of the femur, high in the groin. The ends of the two cords were crossed outside of the leg, both posteriorly and anteriorly, and tied tightly, with the femur acting as an anchor. The isolated femoral vessels and nerve were excluded from this tourniquet. Circulation to the paw was excluded by another tourniquet at the ankle. With these measures, ;95% of the effluent blood flow in this preparation can be attributed to muscle [1]. To prevent collateral arterial flow to the hindlimb, the left deep circumflex and internal and external iliac arteries were ligated through a midline abdominal incision. Before ligation of these vessels, the femoral artery of the left leg was perfused from the controlateral femoral artery. Arterial isolation and reactive hyperemia were documented to be present in all animals at the beginning of each experiment by occluding the femoral artery for 30 s. Heparin was given intravenously at a dose of 1,000 U/kg before cross perfusion was initiated. Blood flow from the left femoral vein was returned to a reservoir positioned above, and connected to, the right femoral vein. After each experiment, the left femoral artery was injected with India ink, and the muscle that stained black was dissected free and weighed. Leg blood flow, DO_2_, and VO_2_ were reported per kilogram of muscle mass.

A roller occlusive pump directed blood flow from the right hindlimb femoral artery to the femoral artery of the vascularly isolated left hindlimb. A sampling port and pressure transducer were placed in this circuit proximal to the limb. A membrane oxygenator (model 0800–2A, Sci Med) was interposed in the perfusion circuit. A gas flow mixer (model GF-3, Cameron Instruments) supplied O_2_, N_2_, and CO_2_ to the oxygenator, as needed, to produce normoxia or hypoxia with normocapnia in the blood supply to the hindlimb. A water bath warmed the oxygenator so that perfusion to the isolated hindlimb was at 37°C after heat loss through the tubing. After the hindlimb preparation was complete, 20 mg of succinylcholine chloride was given intramuscularly and a continuous infusion of 0.1 mg.ml^-1^.min^-1^ was begun. Mechanical ventilation was started at 10 breaths/min with a Harvard animal respirator. Tidal volume was varied to keep systemic arterial PCO_2_ between 30 and 35 mmHg. Anesthetic state was checked periodically by vigorous toe pinching. If systemic blood pressure or heart rate responded, additional anesthetic was given.

***Measurements***

VO_2_ and CO_2_ production were continuously calculated from respiratory volumes and gas fractions by an on-line computer using appropriate analyzers. Expired gas was routed from the animal to a 2-liter mixing chamber and, finally, to a dry gas meter (Harvard Apparatus, Dover, MA) for determination of minute ventilation. Gas fractions were measured by continuous sampling of the mixing chamber with O_2_ and CO_2_ analyzers (S-3a and CD-4, respectively, Applied Electrochemistry, Pittsburgh, PA). The sampled gases were returned downstream to the dry gas meter so that no volume was lost.

Blood samples from the carotid, femoral, and pulmonary arteries and femoral vein were obtained simultaneously. Blood gas tensions and pH were measured in an acid-base analyzer (ABL-30, Radiometer, Westlake, OH) at 37°C and later corrected to esophageal temperature at the time of sampling. Arterial oxygen content was calculated as CaO_2_ (mL) = 1.34 × Hb (g/dL) × SaO_2_ + 0.0031 × PaO_2_ (mmHg), where SaO_2_ is the oxygen saturation of arterial blood, Hb the hemoglobin concentration, and PaO_2_ the arterial oxygen tension. Hindlimb venous oxygen content was calculated as CvO_2_ (mL) = 1.34 × Hb (g/dL) × SvO_2_ + 0.0031 × PvO_2_ (mmHg), where PvO_2_ is the hindlimb venous oxygen tension, and SvO_2_ is the hindlimb venous oxygen saturation. Oxygen saturation was measured with a co-oximeter calibrated for dog blood (IL-282, Instrumentation Lab, Lexing ton, MA).

Cardiac output was calculated by dividing whole body VO_2_ by the difference in CaO_2_ and CvO_2_. All values were reported per unit of body weight.

ΔO_2_ was calculated as CaO_2_ – CvO_2_. Hindlimb VO_2_ was calculated as the product of Q (leg blood flow) and ΔO_2_. Hindlimb oxygen delivery (DO_2_) was calculated by using the formula: DO_2_ (mL/min) = CaO_2_ × Q × 10. Hindlimb oxygen extraction (OE) was defined as: OE= VO_2_/DO_2_.

∆PCO_2_ was calculated as the difference between the hindlimb venous carbon dioxide tension (PvCO_2_) and PaCO_2_. ΔpH was calculated as the difference between arterial pH (pHa) and hindlimb venous pH (pHv). In the original study, the hindlimb difference between venous-to-arterial CO_2_ content (CvCO_2_−CaCO_2_) was calculated with the McHardy equation (as proposed by Neviere et al. [12]): ΔCCO_2_=11.02×[(PvCO_2_)^0.396^−(PaCO_2_)^0.396^] − (15−Hb)× 0.015×(PvCO_2_ − PaCO_2_) − (95 − SaO_2_)× 0.064. However, the most used equation to calculate the blood CO_2_ content is the Douglas equation [13], which includes pH:

Blood CO_2D_ content [blood Douglas CCO_2_ (ml)] =

Plasma CCO_2_ × [1 – 0.0289 × (Hb)/(3.352 – 0.456 × SO_2_) × (8.142 – pH)]

where plasma CCO_2_ = 2.226 × S × plasma PCO_2_ × (1 + 10^pH – pK’^), CCO_2_ is CO_2_ content, SO_2_ is oxygen saturation, S is the plasma CO_2_ solubility coefficient, and pK’ is the apparent pK.

S and pK’ were calculated as follow:

S = 0.0307 + [0.00057 × (37 – T)] + [0.00002 × (37 – T)^2^]

and

pK’ = 6.086 + [0.042 × (7.4 – pH)] + ((38 – T) × {0.00472 + [0.00139 × (7.4 – pH)]})

where T is the temperature expressed as ^0^C.

The difference between venous-to-arterial CO_2_ content calculated with the Douglas equation was: ΔCCO_2D_ = CvCO_2D_ −CaCO_2D_.

To investigate the metabolic acidosis and Haldane effects on the PCO_2_/CCO_2_ relationship, default (Def) values of blood CCO_2_ were calculated with the Douglas’s equation by using only the resting values of pH and SvO_2_ for each dog as following: DefpH-ΔCCO_2D_= DefpH-CvCO_2D_ − DefpH-CaCO_2D_, and DSvO_2_-ΔCCO_2D_= DefSvO_2_-CvCO_2D_ − DefSvO_2­_-CaCO_2D_

Leg blood flow, DO_2_, and VO_2_ were reported per kilogram of muscle mass.

We also calculated the hindlimb ΔPCO_2_/ΔO_2_, ΔCCO_2_/ΔO_2_, and ΔCCO_2D_/ΔO_2_ ratios_._

***Experimental protocol***

After all pressures and flows were stable for at least 30 min, the experiment began with a 30-min control period, during which measurements were obtained every 15 min. In the progressive ischemic hypoxia (IH) group, Q was then decreased every 15 min to produce Q values of 60, 45, 40, 30, 20, 15, and 10 mlzkg21 zmin21. In the hypoxic hypoxia (HH) group, Q was set at 60 mg.kg^-1^.min^-1^ and limb DO_2_ was reduced by decreasing arterial PO_2_ from 100 to 15 mmHg (i.e., CaO2 of 17 to 2 ml O2/100 ml) in eight steps at 15-min intervals. A flow rate of 60 ml.kg^-1^.min^-1^ was chosen for progressive hypoxia because it is within the range of resting blood flow to normal skeletal muscle and for the practical reason that a moderate flow was necessary to achieve the desired low PO_2_ values using the membrane oxygenator. PaCO_2_, PvCO_2_, CaO_2_, CvO_2_, arterial pH (pHa), and venous pH (pHv) were determined every 15 min, 13 min after the change in hindlimb arterial flow or PO_2_.

For each experiment, regression lines were fitted to the delivery-independent and -dependent portions of the delivery-uptake curve using a dual-line, least squares method [2]. The intercept of these two lines defined the critical DO_2_ (DO_2_crit), that is, the delivery at which VO_2_ began to fall with any further decline in DO_2_.

**Statistical analysis**

All data are expressed as mean ± SEM after assessed for normality using the Kolmogorov–Smirnov test.

Comparisons of data within and between groups were performed using a mixed ANOVA. Post-hoc paired and unpaired *t*-tests were used, as appropriate, for one-time comparisons. The Bonferroni method was used to adjust for multiple comparisons.

Statistical analysis was performed using GraphPad Prism 6.0 software for windows (San Diego, California, USA). *P* < 0.006 and *P* < 0.007 were considered statistically significant for the between-group and within-group (with the baseline) comparisons, respectively. All reported *P* values are two-sided.

**Results**

Systemic hemodynamics and oxygen-derived variables remain unchanged throughout the protocol with no differences between the IH and HH models (Supplementary-Table S1).

In both groups, the VO_2_/DO_2_ graph depicts the typical biphasic relationship (Supplementary-Figure S1). There was no statistically significant difference between the mean DO_2Crit_ in the HH and IH models (6.9 ± 0.6 vs. 6.0 ± 0.5 mL/kg/min, p=0.28, respectively). SvO_2_ at DO_2Crit_ was not statistically different between the two groups (25 ± 1.7% in HH vs. 26 ± 1.5% in IH, p=0.66). However, for the lower DO_2_ values, SvO_2_ was significantly higher in the IH model than in the HH group (Supplementary-Figure S2). EO_2_ at DO_2Crit_ was significantly higher in the IH group than in the HH model (74 ± 2 % vs. 60± 4%, p=0.01) and increased continuously and similarly in both groups (Supplementary-Figure S3). ΔPCO_2_ risen significantly in the IH model and did not change in the HH model (Supplementary-Figure S4).

**References**

1. Cain SM and Chapler CK. Oxygen extraction by canine hindlimb during hypoxic hypoxia. J Appl Physiol 46: 1023–1028, 1979.
2. Samsel R and Schumacker PT: Determination of the critical O_2_ delivery from experimental data: sensitivity to error. *J Appl Physiol 1988;* 64: 2074–2082
