# Supplementary material for: Ratio of venous-to-arterial PCO2 to arteriovenous oxygen content difference during regional ischemic or hypoxic hypoxia
Source: Sci Rep. 2021 May 13;11:10172. doi: 10.1038/s41598-021-89703-5 (PMC8119496; doi:10.1038/s41598-021-89703-5)
Supplement: Supplementary file 2 — Supplementary Information 2. [file 41598_2021_89703_MOESM2_ESM.docx]

**Supplemental Digital Content 2**

**Table S1.** Systemic hemodynamics and oxygen-derived variables

| Number of dogs | 12 |
| --- | --- |
| Cardiac output, mL.kg^-1^.min^-1^ | 136 ± 6 |
| Arterial oxygen pressure, mmHg | 82 ± 2 |
| Arterial carbon dioxide pressure, mmHg | 34 ± 2 |
| Mean arterial pressure, mmHg | 128 ± 2 |
| Oxygen consumption, mL.kg^-1^.min^-1^ | 6.67 ± 0.07 |
| Hematocrit at baseline, % | 39.0 ± 0.4 |
| Hematocrit at the end of the study, % | 38.7 ± 0.5 |

Data are presented as mean ± SEM
