# Supplementary material for: Ratio of venous-to-arterial PCO2 to arteriovenous oxygen content difference during regional ischemic or hypoxic hypoxia
Source: Sci Rep. 2021 May 13;11:10172. doi: 10.1038/s41598-021-89703-5 (PMC8119496; doi:10.1038/s41598-021-89703-5)
Supplement: Supplementary file 3 — Supplementary Information 3. [file 41598_2021_89703_MOESM3_ESM.docx]

**Supplemental Digital Content 3**

**Figure S1.** Hindlimb oxygen consumption (VO_2_) as a function of hindlimb oxygen delivery (DO_2_) for ischemic hypoxia model (IH) and hypoxic hypoxia model (HH). There was no statistically significant difference at any DO_2_. Critical DO_2_ (DO_2_crit) was not statistically different in HH (6.9±0.6 ml/kg/min) and IH (6.0±0.5 ml/kg/min).
