# Supplementary material for: Ratio of venous-to-arterial PCO2 to arteriovenous oxygen content difference during regional ischemic or hypoxic hypoxia
Source: Sci Rep. 2021 May 13;11:10172. doi: 10.1038/s41598-021-89703-5 (PMC8119496; doi:10.1038/s41598-021-89703-5)
Supplement: Supplementary file 8 — Supplementary Information 8. [file 41598_2021_89703_MOESM8_ESM.docx]

**Supplemental Digital Content 8**

**Figure S6.** Hindlimb venous CO_2_ content (CCO_2_) as a function of hindlimb venous PCO_2_ for CCO_2_ calculated with accounting for venous oxygen saturation (SvO_2_) changes (with_SvO_2_) and without accounting for SvO_2_ changes (without_SvO_2_) using Douglas equation.
