# Supplementary material for: Ratio of venous-to-arterial PCO2 to arteriovenous oxygen content difference during regional ischemic or hypoxic hypoxia
Source: Sci Rep. 2021 May 13;11:10172. doi: 10.1038/s41598-021-89703-5 (PMC8119496; doi:10.1038/s41598-021-89703-5)
Supplement: Supplementary file 9 — Supplementary Information 9. [file 41598_2021_89703_MOESM9_ESM.docx]

**Supplemental Digital Content 9**

**Figure S7.** Hindlimb arterial-to-venous O_2_ difference (ΔO_2_) as a function of hindlimb oxygen delivery (DO_2_) for ischemic hypoxia model (IH) and hypoxic hypoxia model (HH). **P* < 0.006 vs. HH, ^#^*P* < 0.007 vs. baseline, mixed ANOVA.
