# Supplementary material for: Ratio of venous-to-arterial PCO2 to arteriovenous oxygen content difference during regional ischemic or hypoxic hypoxia
Source: Sci Rep. 2021 May 13;11:10172. doi: 10.1038/s41598-021-89703-5 (PMC8119496; doi:10.1038/s41598-021-89703-5)
Supplement: Supplementary file 11 — Supplementary Information 11. [file 41598_2021_89703_MOESM11_ESM.docx]

**Supplemental Digital Content 11**

**Figure S9.** Hindlimb venous-to-arterial CO_2_ content difference calculated with Douglas equation without accounting for pH changes (DefpH-ΔCCO_2D_) over the arterial-to-venous O_2_ difference (ΔO_2_) ratio (DefpH-ΔCCO_2D_/ΔO_2_) as a function of hindlimb oxygen delivery (DO_2_) for ischemic hypoxia model (IH) and hypoxic hypoxia model (HH). **P* < 0.006 vs. HH, ^#^*P* < 0.007 vs. baseline, mixed ANOVA.
